# Effect of tea polyphenols on the fermentation quality, protein preservation, antioxidant capacity and bacterial community of stylo silage

**DOI:** 10.3389/fmicb.2022.993750

**Published:** 2022-09-08

**Authors:** Yinchao Huang, Chenchen Qiu, Yimin Wang, Wei Zhang, Liwen He

**Affiliations:** State Key Laboratory of Animal Nutrition, College of Animal Science and Technology, China Agricultural University, Beijing, China

**Keywords:** antioxidant activity, bacterial community, fermentation quality, protein preservation, tea polyphenol

## Abstract

The present study was aimed to evaluate the improvement potential of tea polyphenols (**TPP**) on silage characteristics and bacterial community. Stylo ensiled with TPP (0, 0.2 or 0.4%, on a fresh basis) were analyzed for fermentation parameter, protein fraction, antioxidant activity and bacterial community after 7, 14, 30 and 60 days fermentation. The addition of TPP resulted in the decrease (*P* < 0.05) of pH values (5.09 vs. 4.91), dry matter loss (11.77 vs. 8.02% DM), butyric acid concentration (1.64 vs. 1.02% DM) and ammonia-N proportion (13.69 vs. 8.98% CP, on Day 30) of stylo silage as well as the increase (*P* < 0.01) of lactic acid bacteria population (6.17 vs. 7.54 cfu/g FM) and true protein content (6.03 vs. 7.44% DM), particularly at the first 30 days of ensiling. It somewhat enhanced the antioxidant capacity of style silage at the early stage, and altered the bacterial community of stylo silage, with *Clostridium_sensu_stricto_1* and *Lachnoclostridium_5* much decreased but *Enterobacter* and *Clostridium* still being the dominant genera. It is suggested that TPP could help improve fermentation quality and nutrient preservation of stylo silage, and delay proteolysis process and antioxidant decay.

## Introduction

Tea is one of the most popular beverages worldwide, and proper drinking of tea (especially green tea) could provide diverse health benefits in diabetes, cardiovascular and neurological diseases (Juneja et al., 2013; Xing et al., 2019; Guo et al., 2021). Such benefits of tea consumption are generally owed to the presence of kinds of polyphenols, collectively termed as tea polyphenols (**TPP**), which could account for 24–36% in dry weight of green tea (Juneja et al., 2013; Xing et al., 2019). With the increasing awareness and fashion of drinking tea, various kinds of ready-made tea drinks are produced and meanwhile quantities of tea grounds are released as the main byproduct (Wang and Xu, 2014; Guo et al., 2021). Additionally, a large amount of green tea waste is also produced due to pruned tea branches and the discard of unqualified part during tea processing. Generally, tea plant is rich in tannins, saponins, proteins, amino acids, lipids, sugars, vitamins and minerals (Ramdani et al., 2013). Numerous studies suggest that dietary inclusion of tea grounds or tea waste would contribute to the improvement of nutrient utilization, growth performance, meat quality, immune response, methane emission (Xu et al., 2008; Nasehi et al., 2018; Alagawany et al., 2019), which are believed to correlate with its multiple functional components (e.g., polyphenols, theophylline) and abundant nutrients (Kondo et al., 2014; Xing et al., 2019). The use of such tea byproducts as a feedstuff in ruminant nutrition can be beneficial for both economical and environmental reasons (Nasehi et al., 2018; Guo et al., 2021).

Due to the potential activities of antioxidant, anti-inflammation, anti-microbial, anti-fungal, TPP has been well recognized in industries, especially as a natural source of food antioxidants (Alagawany et al., 2019; Khan and Mukhtar, 2019; Xing et al., 2019). Likewise, proper amount of tea bioactive extracts as dietary supplements can improve the growth performance, muscle and meat quality, antimutagenic and other characteristics of animals (Cimmino et al., 2018; Guo et al., 2021). The addition of green tea waste improves the lactic acid bacteria growth and fermentation during ensiling, especially when the mixture forage materials are not insufficient for lactic acid production (Kondo et al., 2006; Guo et al., 2021). Moreover, the study of Han and Zhou (2013) shows that TPP could also inhibit fatty acids oxidation in corn silage mainly by inhibiting lipoxygenases activity, and decrease ammonia-N concentration. Other than strong antioxidant activity, TPP also possess a great protein binding ability endowed by its polyhydroxyl structure, which might help prevent protein from hydrolyzing during ensiling. From the above, it is believed that TPP owes the potential to improve silage fermentation and quality as a novel additive. However, the results of Liu et al. (2016, 2018) indicate that TPP application is not effective enough in preventing fatty acids oxidation in high moisture alfalfa silage during ensiling and after exposure to air, neither effective in reducing alpha-tocopherol and beta-carotene loss in high moisture napier grass silage. Such discrepancies of TPP application might be due to the differences of silage material, inclusion level and storage condition. Further investigations are still needed to justify the effectiveness of TPP as a silage additive. To date, no research has been reported to investigate the effect of TPP on the succession of bacterial community during ensiling fermentation.

Thus, this study was to investigate the effectiveness of TPP application in improving silage fermentation, particularly paying attention to the dynamic changes of ensiling characteristics, nitrogen fractions, antioxidant capacity and bacterial community of stylo silage. The results would give us a whole evaluation of TPP as a novel additive in silage production, providing an instruction for TPP or tea residue utilization.

## Materials and methods

### Raw materials and silage preparation

Fresh stylo (*CIAT 184*) was mowed at early bloom stage using a sickle, with a 5 cm stubble left, and then manually chopped into 1–2 cm by cutter. Subsequently, the forage was ensiled without (**Control**) or with addition of 0.2% (**TPP1**) and 0.4% (**TPP2**) tea polyphenols (CAS 84650-60-2, powder reagent with a purity of 99%; Shanghai McLean Biochemical Technology Co., Ltd, China) on a fresh matter (**FM**) basis. As for the chemical composition of the TPP reagent, the merchandise instruction read as catechin accounting for 60–80%, mainly covering epigallocatechin gallate (EGCG), epigallocatechin (EGC), epicatechin (EC) and epicatechin gallate (ECG). A given weight of TPP powder was resolved in distilled water and evenly dispensed using a mini sprayer. In total, 36 lab-level silage bags (20 cm by 30 cm in size, 200 g per bag) were individually prepared (3 treatments × 4 sampling points × 3 replicates) and sealed with a household vacuum sealer (Lvye DZ280; Dongguan Yijian Packaging Machinery Co. Ltd, China). After 7, 14, 30 and 60 days fermentation at natural temperature (25–30°C), 3 silo bags of each treatment were randomly unsealed to sample for the analyses of fermentation parameter, nitrogen fraction, antioxidant capacity and microbial community.

### Determination of microbial population, fermentation parameter, nitrogen fraction and antioxidant capacity

According to the procedures detailed by He et al. (2019c), lactic acid bacteria (**LAB**), coliform bacteria, yeast and mold were cultivated with Man Rogosa Sharpe (**MRS**), Violet Red Bile and Rose Bengal agar, respectively. The pH value of silage extract liquid was measured by a glass electrode pH meter (PHS-3C, INESA Scientific Instrument Co. Ltd, Shanghai, China). Ammonia-N content was measured in colorimetry (Broderick and Kang, 1980). The concentrations of organic acids (lactic acid, acetic acid, propionic acid, butyric acid) were analyzed using high performance liquid chromatography (**HPLC**). Another silage sample was lyophilized to measure radical scavenging activity (**DPPH** and **ABTS**) and ferric reducing antioxidant power (**FRAP**) in colorimetrical methods. Finally, the remaining silage was oven-dried at 65°C for 48 h to determine dry matter (**DM**), then ground (1 mm sieve) for the analysis of nitrogen fractions (crude protein, true protein and nonprotein-N) using an automatic Kjeldahl apparatus (Kjeltec 8400, Foss Analytics, Hillerød, Denmark), where true protein was measured according to the method of Licitra et al. (1996), and nonprotein-N content was calculated by the difference of crude protein and true protein. The content of water soluble carbohydrate (**WSC**) in the raw material was also analyzed with 3, 5-dinitrosalicylic acid colorimetric method (The Ministry of Agriculture (MOA), 2015). Additionally, each bag was individually weighed at ensiling time and sampling time to monitor dry matter loss (**DML**).

### Sequencing analysis of bacterial community

The DNA extraction and sequencing analysis were conducted on the platform of Gene Denovo Co. Ltd. (Guangzhou, China). In brief, DNA extraction was conducted using commercial DNA Kit (Omega Biotek, Norcross, GA, US) and PCR amplification was conducted with the primers (341F: CCTACGGGNGGCWGCAG; 806R: GGACTACHVGGGTATCTAAT) targeting at the V3-V4 region of 16S rDNA. Following purification and quantification, the amplicons were sequenced on Illumina Hiseq 2500. The sequencing data were processed with the procedures stated in Wang et al. (2019). In brief, paired-end clean reads were merged using FLSAH (version 1.2.11) and noisy sequences were filtered with QIIME (version 1.9.1), and then chimera checking was performed using UCHIME algorithm. Operational taxonomic unit (**OTU**) with 97% identities was generated using UPARSE pipeline, and taxonomy assignment was performed using Ribosome Database Project (**RDP**) classifier (Version 2.2). The alpha diversity indices containing Sobs, Shannon, Simpson, Chao and Ace were calculated in QIIME (version 2.15.3). The abundance statistics of each taxonomy was visualized using Krona (version 2.6). The stacked bar plot of the community composition was visualized in R project ggplot2 package (version 2.2.1), so did principal coordinates analysis (**PCoA**). The sequencing data reported in this study was archived in the Sequence Read Archive (**SRA**) with the accession number PRJNA 734171.

### Statistical analysis

The effects of TPP dose, ensiling duration and their interaction were analyzed using general linear model (**GLM**) procedure of SAS 9.3 (SAS Institute Inc., Cary, NC, USA), and Duncan's multiple comparisons were performed with significance declared at the level of *P* < 0.05. The sequencing analysis was performed on OmicShare platform (http://www.omicshare.com/tools).

## Results

### General characteristics of fresh stylo

General characteristics covering the contents of dry matter, crude protein, true protein, nonprotein-N and WSC, the populations of LAB, yeast, mold and coliform bacteria, and the antioxidant activities of DPPH, ABTS and FRAP of fresh stylo used for silage production are summarized in Table 1. In the present study, fresh stylo had a DM content of 25.63% and a crude protein content of 12.68% DM, of which almost 70% was true protein (8.82% DM). The WSC content was low as 1.45% DM in the raw material. The populations of LAB, yeast, mold and coliform bacteria were 4.03, 4.12, 3.10, 4.89 log_10_ cfu/g FM, respectively.

**Table 1:** General characteristics of fresh stylo used for silage production.

**Item**	**Content**
Dry matter (%)	25.63 ± 0.13
Crude protein (% DM)	12.68 ± 0.58
True protein (% DM)	8.82 ± 0.46
Nonprotein-N (% DM)	3.85 ± 0.55
Water soluble carbohydrate (% DM)	1.45 ± 0.15
Lactic acid bacteria (Log_10_ cfu/g FM)	4.03 ± 0.29
Yeast (Log_10_ cfu/g FM)	4.12 ± 0.19
Mold (Log_10_ cfu/g FM)	3.10 ± 0.17
Coliform bacteria (Log_10_ cfu/g FM)	4.89 ± 0.36

DM, dry matter; FM, fresh matter.

### Fermentation quality of stylo silage ensiled with tea polyphenols addition

The dynamic changes of DM loss, organic acid content and microbial population of stylo silage are showed in Table 2. In the present study, DM content, pH value and microbial population of stylo silage decreased (*P* < 0.01) as ensiling fermentation was prolonged, while DM loss and acetic acid concentration increased (*P* < 0.01), along with lactic acid and butyric acid not detected by 30 days of ensiling. Moreover, the pH values of all stylo silages declined to around 5.0 after 60 days fermentation. As to the effect of treatment, TPP addition resulted in the increase (*P* < 0.01) of DM content and LAB population as well as the decrease (*P* < 0.05) of DM loss, butyric acid and pH value of stylo silage. Additionally, a relative high level of coliform bacteria count (> 5 log cfu/g FM) was found in the first 14 days of ensiling fermentation but all the silages had small populations of yeast and mold (<2 log cfu/g FM).

**Table 2:** Effect of tea polyphenols on the dynamic fermentation of stylo silage.

**Item**	**Treatment**	**Days of ensiling**				**SEM**	***P*** **-value**		
		**7**	**14**	**30**	**60**		**D**	**T**	D*T
DM	Control	25.54^a^	24.52^Bb^	24.17^Bbc^	23.89^B^	0.33	<0.01	<0.01	0.80
	TPP1	26.05^a^	25.40^Aab^	25.09^Ab^	24.95^Ab^				
	TPP2	25.92^a^	25.70^Aa^	25.20^Ab^	24.65^ABc^				
DML	Control	1.39^c^	5.49^Ab^	7.42^Ab^	11.77	1.05	<0.01	0.01	0.64
	TPP1	0.50^d^	2.81^Bc^	5.75^ABb^	8.02^Ba^				
	TPP2	1.37^c^	2.17^Bc^	5.08^Bb^	9.58^Ba^				
pH	Control	5.47^Aa^	5.41^Aa^	5.25^Bb^	5.09	0.03	<0.01	<0.01	<0.01
	TPP1	5.34^Ba^	5.35^ABa^	5.38^Aa^	5.03^Ab^				
	TPP2	5.31^Ba^	5.32^Ba^	5.14^Cb^	4.91^Bc^				
LA	Control	ND	ND	0.45	0.	0.06	-	0.43	-
	TPP1	ND	ND	0.38	0.67				
	TPP2	ND	ND	0.40	0.67				
AA	Control	0.25^c^	0.41^b^	0.61^a^	0.5	0.03	<0.01	0.26	0.94
	TPP1	0.24^c^	0.37^b^	0.58^a^	0.48^ab^				
	TPP2	0.25^c^	0.40^b^	0.58^a^	0.53^a^				
BA	Control	ND	ND	ND	1.64^A^	0.08	-	0.02	-
	TPP1	ND	ND	ND	1.33^B^				
	TPP2	ND	ND	ND	1.02^C^				
LAB	Control	7.91^a^	7.97^a^	6.98^Bb^	6.17	0.14	<0.01	<0.01	<0.01
	TPP1	7.88^a^	8.16^a^	7.34^ABb^	7.30^Ab^				
	TPP2	7.99^a^	7.93^a^	7.75^Aab^	7.54^Aa^				
Yeast	Control	2.33	3.63	<2.00	<2.00				
	TPP1	2.45	2.36	2.15	<2.00	-	-	-	-
	TPP2	2.85	2.74	<2.00	<2.00				
Coliform	Control	6.76	5.38	<2.00	<2.00				
	TPP1	7.27	6.19	3.86	<2.00	-	-	-	-
	TPP2	7.23	5.95	3.85	<2.00				
Mold	Control	<2.00	<2.00	<2.00	<2.00				
	TPP1	<2.00	<2.00	<2.00	<2.00	-	-	-	-
	TPP2	<2.00	<2.00	<2.00	<2.00				

FM, fresh matter; DM (% FM), dry matter; DML (% DM), dry matter loss; LA (% DM), lactic acid; AA (% DM), acetic acid; propionic acid and butyric acid was not detected; LAB (Log_10_ cfu/g FM), lactic acid bacteria; Control, silage ensiled without addition of tea polyphenols; TPP1 and TPP2, silage ensiled with addition of 0.2%, 0.4% tea polyphenols on a FM basis; ND, not detected; SEM, standard error of the means; D, the effect of ensiling days; T, the effect of tea polyphenols; D^*^T, the interaction of ensiling days and tea polyphenols.

Means with different lowercase/uppercase letters in the same row/column differ at *P* < 0.05.

Protein fraction content as well as its corresponding proportion are presented in Table 3. As silage fermentation went on, true protein content of stylo silage decreased (*P* < 0.01), and ammonia-N concentration and its proportion increased (*P* < 0.01). Referring to the treatments, TPP addition led to the increase (*P* < 0.01) of true protein concentration and the decrease (*P* < 0.01) of ammonia-N concentration and proportion in the first 30 days of ensiling fermentation.

**Table 3:** Effect of tea polyphenols on the dynamic protein fraction of stylo silage.

**Item**	**Treatment**	**Days of ensiling**				**SEM**	***P*** **-value**		
		**7**	**14**	**30**	**60**		**D**	**T**	D*T
CP	Control	12.88^AB^	12.05	12.01	11.7	0.38	0.07	0.04	0.09
	TPP1	12.32^B^	12.16	11.65	12.32^AB^				
	TPP2	13.30^A^	12.27	12.14	13.11^A^				
TP	Control	6.87^Ba^	6.83^Ba^	5.98^Bb^	6.03	0.37	0.01	<0.01	0.21
	TPP1	7.10^Bab^	7.65^Aa^	6.73^ABb^	6.61^ABb^				
	TPP2	7.99^A^	7.31^AB^	7.55^A^	7.44^A^				
NPN	Control	6.01	5.22	6.03	5.	0.48	0.36	0.57	0.17
	TPP1	5.22	4.51	4.92	5.70				
	TPP2	5.31	4.96	4.59	5.67				
AN	Control	0.67^c^	1.23^Ab^	1.66^Aa^	1.5	0.11	<0.01	0.01	0.26
	TPP1	0.60^d^	0.80^Bc^	1.23^Bb^	1.58^a^				
	TPP2	0.62^c^	0.74^Bc^	1.09^Bb^	1.64^a^				
TPR	Control	53.36	56.92	50.10	51.	3.12	0.14	0.06	0.23
	TPP1	57.76	62.89	57.77	53.72				
	TPP2	60.07	59.58	62.04	56.86				
ANR	Control	5.20^c^	10.17^Ab^	13.69^Aa^	13.0	0.79	<0.01	<0.01	0.01
	TPP1	4.90^d^	6.63^Bc^	10.56^Bb^	12.82^a^				
	TPP2	4.68^c^	6.04^Bc^	8.98^Cb^	12.51^a^				

CP (% DM), crude protein; TP (% DM), true protein; NPN (% DM), nonprotein nitrogen, expressed as CP equivalent; AN (% DM), ammonia nitrogen, expressed as CP equivalent; TPR (% CP), the ratio of TP and CP; NPNR (% CP), the ratio of NPN and CP; ANR (% CP), the ratio of AN and CP; Control, silage ensiled without addition of tea polyphenols; TPP1 and TPP2, silage ensiled with addition of 0.2%, 0.4% tea polyphenols on a FM basis; SEM, standard error of the means; D, the effect of ensiling days; T, the effect of tea polyphenols; D^*^T, the interaction of ensiling days and tea polyphenols.

Means with different lowercase/uppercase letters in the same row/column differ at *P* < 0.05.

The antioxidant activities of DPPH, ABTS and FRAP of stylo silage are showed in Table 4. In the present study, the activities of DPPH, ABTS and FRAP decreased (*P* < 0.05) as ensiling fermentation was extended. Relative to the control silage, TPP addition of 0.4% led to the increase (*P* < 0.01) of FRAP across the process, along with an increased ABTS in the first 14 days of ensiling fermentation.

**Table 4:** Effect of tea polyphenols on the dynamic antioxidant capacity of stylo silage.

**Item**	**Treatment**	**Days of ensiling**				**SEM**	***P*** **-value**		
		**7**	**14**	**30**	**60**		**D**	**T**	D*T
DPPH	Control	7.27^b^	16.23^a^	4.63^b^	4.07^b^	4.22	0.01	0.17	0.28
	TPP1	15.67^a^	5.40^b^	4.57^b^	2.83^b^				
	TPP2	18.00^ab^	21.87^a^	7.87^bc^	2.63^c^				
ABTS	Control	2.23^Ba^	2.40^Ba^	2.50^a^	1.67^b^	0.35	<0.01	0.03	0.25
	TPP1	2.67^Ba^	2.07^Bab^	1.90^b^	1.47^b^				
	TPP2	3.47^Aa^	3.50^Aa^	2.27^b^	1.67^b^				
FRAP	Control	3.43^B^	3.63^B^	3.60^B^	3.63^B^	0.19	0.02	<0.01	<0.01
	TPP1	3.87^B^	3.63^B^	3.87^B^	3.87^AB^				
	TPP2	4.70^Aa^	4.63^Aa^	4.63^Aa^	4.20^Ab^				

TE, trolox equivalent; DPPH (mg of TE/g of DM), free radical DPPH scavenging activity; ABTS (mg of TE/g of DM), radical ABTS^+^ scavenging activity; FRAP (mg of TE/g of DM), ferric-reducing antioxidant power; Control, silage ensiled without addition of tea polyphenols; TPP1 and TPP2, silage ensiled with addition of 0.2%, 0.4% tea polyphenols on a FM basis; SEM, standard error of the means; D, the effect of ensiling days; T, the effect of tea polyphenols; D^*^T, the interaction of ensiling days and tea polyphenols.

Means with different lowercase/uppercase letters in the same row/column differ at *P* < 0.05.

### Bacterial community of stylo silage ensiled with tea polyphenols addition

**Figure 1:**
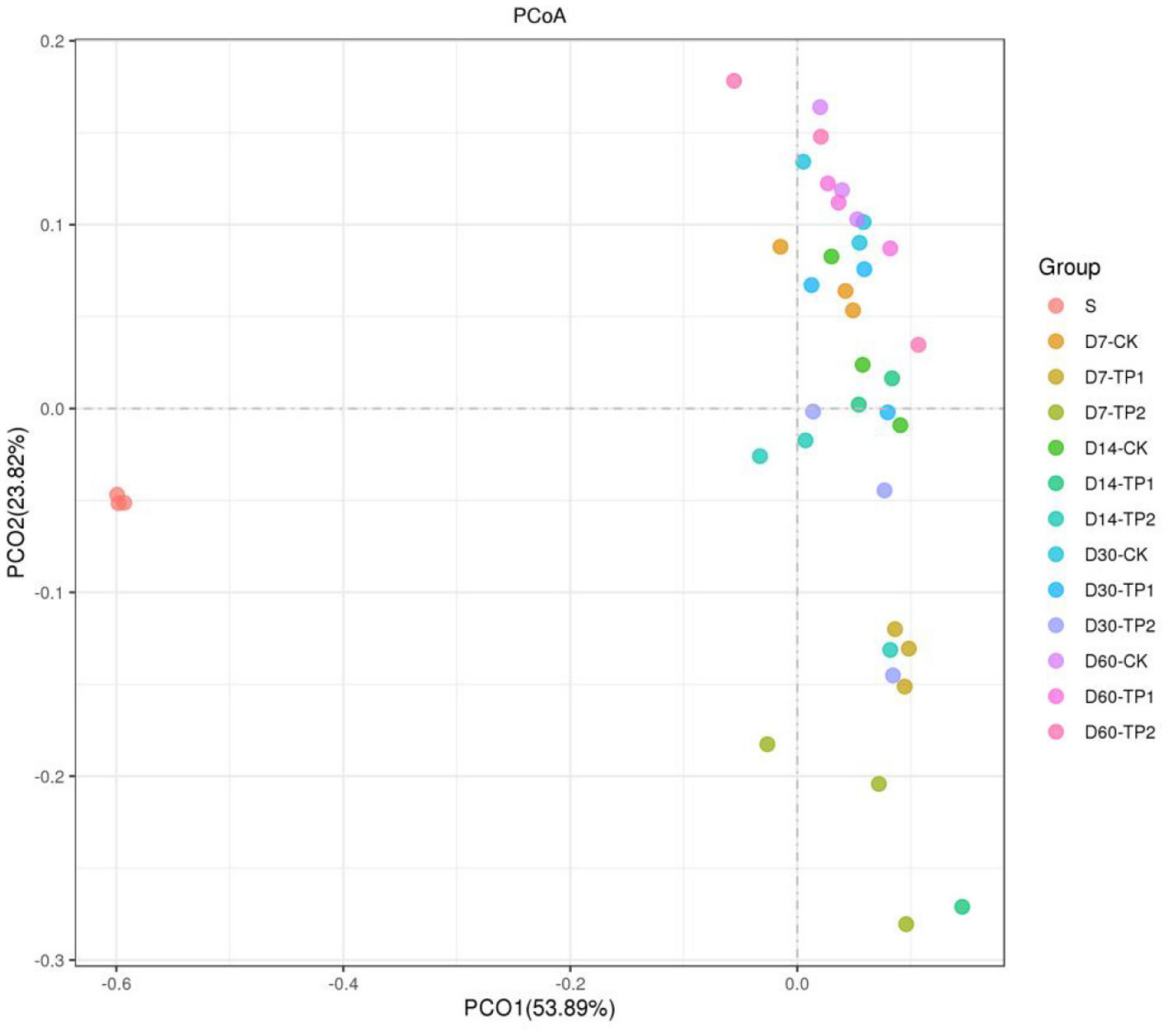
Principal coordinates analysis (**PCoA**, Weighted UniFrac) of bacterial community in the stylo silage ensiled with/without TPP addition (S: raw stylo; D7-CK: on day 7 of ensiling fermentation-blank control, silage ensiled without addition of tea polyphenols; TP1 and TP2: silage ensiled with addition of 0.2%, 0.4% tea polyphenols on a FM basis).

Alpha diversity of bacterial community in stylo silage is listed in Table 5. Sequencing Goods_coverage values of raw material and stylo silages were all over 0.99, and the parameters of Sobs, Shannon, Simpson, Chao and Ace of bacteria in the stylo silages were numerically larger than those of raw material. In comparison, no significant effect (*P* > 0.05) was found on these parameters neither by ensiling duration nor TPP addition, where sobs value tended to decline as ensiling fermentation was extended and the addition of TPP showed an tendency to increase the Simpson value. Principal coordinates analysis (**PCoA**) by weighted uniFrac method clearly illustrated the succession changes of bacterial community as ensiling fermentation was prolonged, but the samples of different groups could not be individually clustered at each timepoint (Figure 1).

**Table 5:** Alpha diversity of bacterial community of stylo silage ensiled with addition of tea polyphenols.

**Item**	**Treatment**	**Days of ensiling**					**SEM**	***P*** **-value**		
		**0**	**7**	**14**	**30**	**60**		**D**	**T**	D*T
Sobs	Control	1,031	1,329	1,309	1,389	1,340	53	0.08	0.55	0.53
	TPP1		1,367	1,407	1,368	1,285				
	TPP2		1,373	1,409	1,485	1,266				
Shannon	Control	1.47	4.80	4.62	4.62	4.24	0.26	0.32	0.13	0.86
	TPP1		4.15	4.23	4.45	4.22				
	TPP2		4.13	4.39	4.42	3.85				
Simpson	Control	0.25	0.86	0.82	0.83	0.81	0.04	0.87	0.06	0.69
	TPP1		0.75	0.74	0.82	0.81				
	TPP2		0.73	0.79	0.77	0.76				
Chao	Control	1,501	2,330	2,299	2,408	2,263	86	0.18	0.81	0.54
	TPP1		2,280	2,428	2,322	2,253				
	TPP2		2,496	2,351	2,393	2,190				
Ace	Control	1,561	2,390	2,374	2,480	2,352	81	0.19	0.99	0.32
	TPP1		2,321	2,603	2,376	2,303				
	TPP2		2,460	2,398	2,458	2,304				
Goods_ coverage	Control	1.00	0.99	0.99	0.99	0.99				
	TPP1		0.99	0.99	0.99	0.99	-	-	-	-
	TPP2		0.99	0.99	0.99	0.99				

RS, raw stylo; Control, silage ensiled without addition of tea polyphenols; TPP1 and TPP2, silage ensiled with addition of 0.2%, 0.4% tea polyphenols on a FM basis.

**Figure 2:**
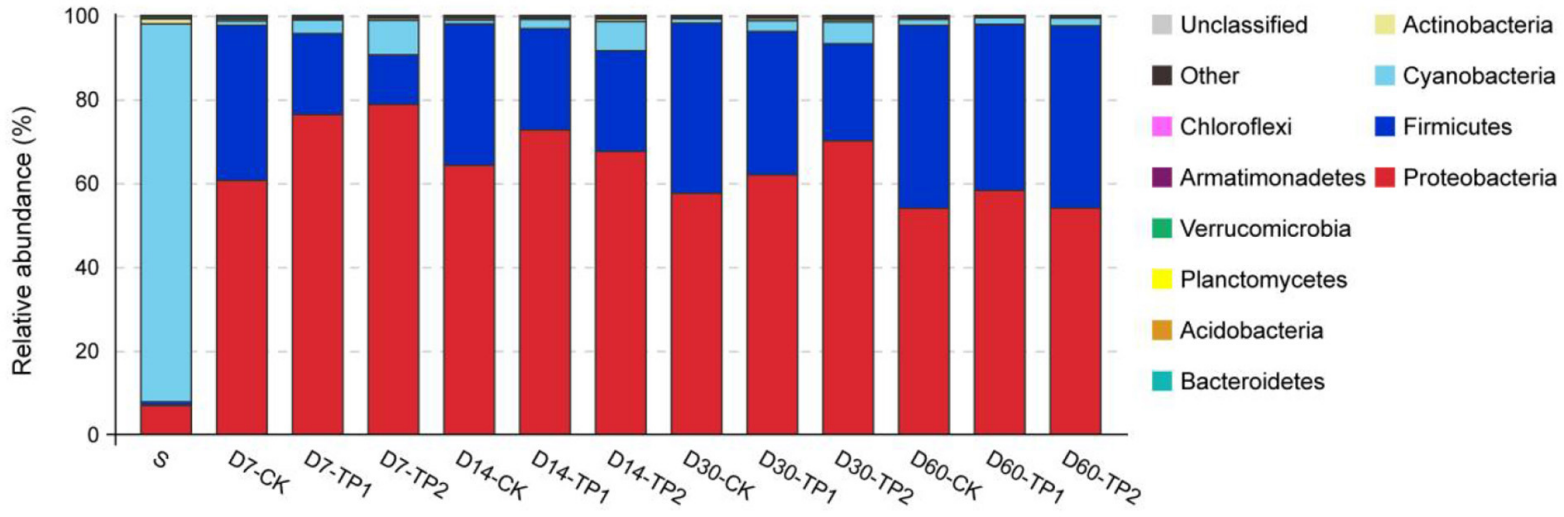
Relative abundance of bacterial community on phylum level of stylo silage ensiled with addition of tea polyphenols (S: raw stylo; D7-CK: on day 7 of ensiling fermentation-blank control, silage ensiled without addition of tea polyphenols; TP1 and TP2: silage ensiled with addition of 0.2%, 0.4% tea polyphenols on a FM basis).

The relative abundance of bacterial community on phylum level is illustrated in Figure 2. Cyanobacteria dominated (relative abundance of 90.20%) in raw stylo while Proteobacteria (53.98–78.79%) and Firmicutes (11.79–43.62%) were the two dominant phyla in stylo silage. In comparison, ensiling duration exerted an effect (*P* < 0.05) on the relative abundance of phyla Proteobacteria, Firmicutes, Cyanobacteria, Planctomycetes, Verrucomicrobia and Armatimonadetes, of which the abundance of Firmicutes increased and those of the others declined as ensiling fermentation was extended. On the contrary, the abundance of Firmicutes was increased (*P* < 0.01) and those of Proteobacteria, Cyanobacteria, Verrucomicrobi were decreased (*P* < 0.05) by the addition of TPP, particularly TPP2.

**Figure 3:**
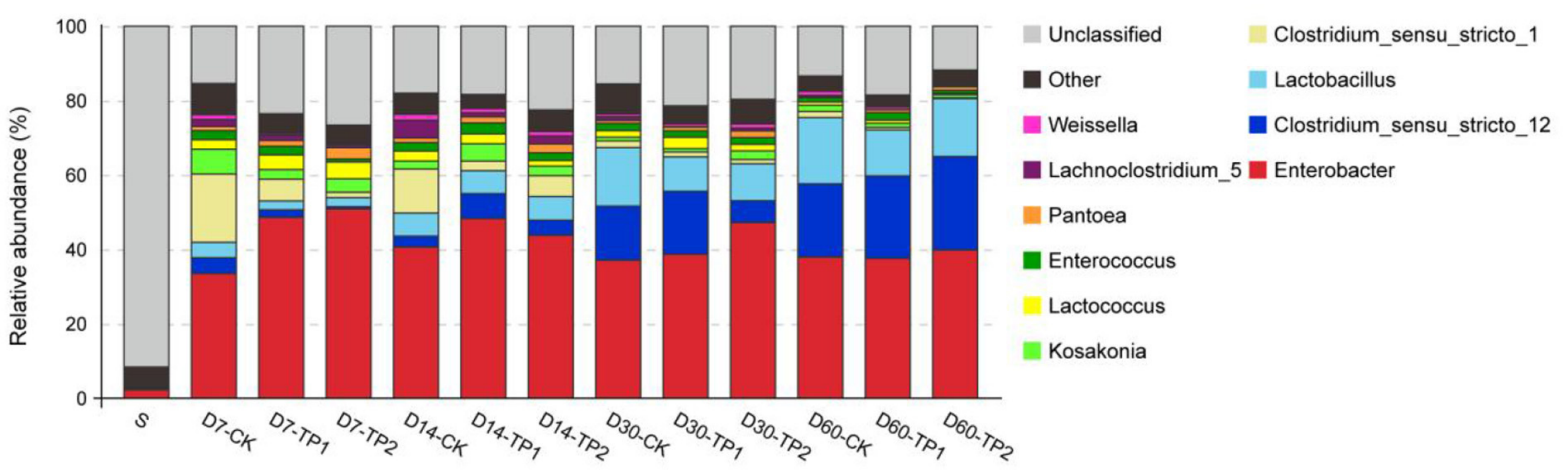
Relative abundance of bacterial community on genus level of stylo silage ensiled with addition of tea polyphenols (S: raw stylo; D7-CK: on day 7 of ensiling fermentation-blank control, silage ensiled without addition of tea polyphenols; TP1 and TP2: silage ensiled with addition of 0.2%, 0.4% tea polyphenols on a FM basis).

As the abundance stacked bar on genus level showed in Figure 3, unclassified bacteria accounted for 91.60% of bacterial community in raw stylo, whereas only 11.81–26.61% of bacterial community was unclassified in stylo silage. In general, *Enterobacter* (33.53–50.88%) and *Clostridium* (2.09–25.31%) were the two dominant genera across the ensiling process, while common silage LAB like *Lactobacillus* (2.41–17.80%), *Lactococcus* (0.44–4.54%), *Enterococcus* (0.80–2.96%), *Weissella* (0.38–1.62%) had a low abundance. Regardless of TPP influence, the abundance of *Clostridium_sensu_stricto_*12 and *Lactobacillus* increased (*P* < 0.01) while those of *Clostridium_sensu_stricto_1, Kosakonia, Lactococcus, Pantoea* and *Lachnoclostridium_5* decreased (*P* < 0.05) as ensiling fermentation went on. After a 60-day fermentation period, the top three genera in the stylo silage ranked as *Enterobacter, Clostridium_sensu_stricto_*12 and *Lactobacillus*. In comparison, TPP addition led to the decrease (*P* < 0.05) of the relative abundance of *Lactobacillus, Clostridium_sensu_stricto_1* and *Lachnoclostridium_5*, along increased *Pantoea* abundance (*P* < 0.05). As the common LAB in silage, the total relative abundance of *Lactobacillus, Lactococcus, Enterococcus* and *Weissella* on day 30 or 60 of ensiling was much lower (*P* < 0.05) in TPP-treated silage (14.67, 14.62; 15.89, and 17.25%) relative to the control (20.10%; 20.96%).

## Discussion

### General characteristics of fresh stylo

Moisture content of raw material is a critical factor determining silage fermentation quality. It is reported that the ideal DM content for ensiling is 30–35% given that a low DM level would lead to a high clostridial activity and a large effluence loss while a high DM would be not beneficial to silage compaction or microbial activity (Yitbarek and Tamir, 2014; Guyader et al., 2018). In the present study, fresh stylo had a low DM content, close to the reported values (He et al., 2019a,b), and a relatively low number of epiphytic LAB along with considerable population of undesirable microorganisms, which might be not beneficial to the dominance establishment of LAB and a rapid pH decline. As a leguminous plant, stylo is a quality forage with medium protein level for ruminants, particularly in tropical and subtropical regions. The preservation of protein during ensiling process would remarkably influence its nutritional value in that nonprotein-N is inferior in utilization efficiency in ruminant nutrition when compared to true protein (Mcdonald et al., 1991; He et al., 2019b). Other than nutrient supply, fresh forage might be also a source of antioxidant activity, which might promote animal health and production. However, a low level of antioxidant activity was found in the fresh stylo, which might be owed to its poor quality, with dry stem and rare leaves.

### Fermentation quality of stylo silage ensiled with tea polyphenols addition

As a general rule of thumb, pH is a comprehensive indicator reflecting the quality of ensiling fermentation. Silage pH is mainly dictated by the generation of lactate and its intrinsic buffering capacity (Kung et al., 2018). In the present study, pH value of stylo silage experienced a slight decline during ensiling process and that of mature silage was around pH 5.0, which is apparently higher than that of typical pH (4.3-4.5) of quality legume silage (Kung et al., 2018). Such a high pH value and its limited decline were likely ascribed to the high buffering capacity and the low acid yield. Even though high LAB population (6.17–8.16 log_10_ cfu/g FM) existed, the concentration of lactic acid and acetic acid in the silage was very low, even undetected at the early stage of ensiling fermentation. Similarly, the study of Kondo et al. (2014) also shows that lactic acid concentration is low in tea grounds silage along with high LAB count (6.28 log_10_ cfu/g FM). Maybe the produced lactic acid was rapidly metabolized by some microorganisms (e.g., *Acetobacter*). The exact reason deserves further research. What's worse, it is undesired that a high level of butyric acid content was found in the mature silage by 60 days of ensiling fermentation. It is reported that butyric acid > 0.5% DM in ration would result in reduced feed intake and other metabolic diseases (Muck, 2010). Consequently, such poor fermentation quality came with a considerable DM loss (11.7% DM) during ensiling process, resulting in low nutritional value of the mature silage. Referring to the treatments, TPP addition led to the increase of LAB population as well as the decrease of pH value, butyric acid content and DM loss in stylo silage. It might be due to the antimicrobial activity of TPP, which would alter the competition between lactic acid bacteria and undesirable microorganisms. The inhibition of undesirable bacteria like *Clostridium butyricum* would likely promote the growth of LAB and nutrient preservation. However, the decline of pH value is not low enough mainly due to the low WSC content in the raw material as well as the low dose of TPP (the higher TPP level was slightly better).

Extensive protein was hydrolyzed in stylo silage during ensiling process, in line with many previous reported results (He et al., 2019a,b, 2021). With the addition of TPP, protein preservation of the silage was enhanced in that the increase of true protein content and the decrease of ammonia-N proportion, particularly at the early stage of ensiling fermentation. In other words, TPP addition resulted in the delay of true protein degradation. It might be interpreted as, TPP possess a great protein binding ability endowed by the polyhydroxyl structure, which would protect protein from enzymatic hydrolysis or inactivate enzymes by generating TPP-protein/enzymes complex, resulting in less protein degraded into nonprotein-N. Moreover, the lower pH value resulting from TPP addition would likely lower the activity of plant proteases, which is believed to initiate proteolysis in silage (Li et al., 2018). Meanwhile, the deamination activity of microorganisms like *Clostridium* and *Enterobacter* might be also inhibited by the lower pH and the antimicrobial activity of TPP. Similarly, kinds of tannins have been proved to help protein preservation in silages (He et al., 2019a; Jayanegara et al., 2019). However, the improvement effect of TPP addition on protein preservation almost disappeared in the mature silage (by Day 60). It might be due to that the low level of TPP in silage could only lower down but not inactivate the activities of enzymes and microbes, indicating that silage fermentation not reach a steady status.

Additionally, fresh forage usually contains kinds of active components and exhibits various biological activities, which might benefit animals' health and performance. However, stylo silage possessed a low antioxidant activity, which gradually decreased during ensiling process. The degradation of antioxidants like polyphenols, vitamins, amino acids or oligopeptides during ensiling would necessarily result in the decrease of antioxidant (Cohen-Zinder et al., 2017). In comparison, TPP addition slightly enhanced the antioxidant activity at the early stage of ensiling fermentation, inferring that TPP addition led to the delay of antioxidant recession. Moreover, such improvement effect was only found in the higher inclusion level of TPP. This might be ascribed to the intrinsic antioxidant and antimicrobial activities of TPP.

### Bacterial community of stylo silage ensiled with tea polyphenols addition

In principle, the fermentation parameters and nutrient preservation of silage reflect the final result of microbial competition during ensiling process. Throwing light on the bacterial community would help to interpret the silage fermentation. Goods_coverage over 0.99 verified that the sequencing result had a good representation for the bacterial community. Relative to the raw material, alpha diversity of bacterial community apparently increased in the silage. Moreover, TPP addition tended to decrease Simpson values, indicating that the diversity of bacterial community was decreased, especially in the treatment of TPP2. It might be ascribed to the inhibition of bacterial activity by TPP. However, the samples of different groups could not be individually clustered at each timepoint in PCA analysis, even though the succession changes of bacterial community were clear in prolonged silage. It is indicated that bacterial community of stylo silage remarkably changes during ensiling fermentation, and is indeed altered by TPP addition but not remarkable enough.

On the phylum level, Cyanobacteria dominated raw stylo and then was substituted by Proteobacteria and Firmicutes after ensiling fermentation. As Cyanobacteria are photoautotrophic organisms capable of oxygenic photosynthesis and can convert inert atmospheric nitrogen into an organic form, such as nitrate or ammonia, their growth and physiology are affected by light and other factors like nutrients supply, temperature (Carr and Whitton, 1982). Thus, it might be the lack of light in silage inhibiting the bloom of Cyanobacteria. Similarly, Li et al. (2019) reported that Cyanobacteria is the main genus in king grass, paspalum, white popinac and stylo before ensiling but undergoes different changes in their silages. In the present study, the abundance of Firmicutes increased and those of phyla Proteobacteria, Cyanobacteria, Planctomycetes, Verrucomicrobia and Armatimonadetes declined as ensiling fermentation was extended. The study of Ogunade et al. (2018) showed that phylum Firmicutes accounted for ~74% of the bacterial community in alfalfa silage, followed by the phylum Proteobacteria but only with a low abundance (about 1%) of Cyanobacteria. Our another study showed that Cyanobacteria, Firmicutes and Proteobacteria were the top three phyla in *Neolamarckia cadamba* leaves silage (He et al., 2020). Such discrepancy among studies might be due to the differences of raw material feature and ensiling processing. As to the treatment, TPP addition led to the increase of Firmicutes abundance and the decrease of phyla Proteobacteria, Cyanobacteria, Verrucomicrobi. It is suggested that TPP addition makes a difference in the bacterial community of stylo silage on phylum level.

Further analysis of bacterial community on genus level illustrated that, the abundance of unclassified bacteria was much higher in fresh stylo than that in stylo silage. It might be due to the relatively poor development of Cyanobacterial taxonomy, where most of *Cyanobacteria* can't be cultured and characterized in the present knowledge (Palinska and Surosz, 2014). Regardless of TPP addition, *Enterobacter* and *Clostridium* were the dominant genera across the ensiling process. After a 60-day fermentation period, the top three genera in the stylo silage ranked as *Enterobacter, Clostridium_sensu_stricto_*12 and *Lactobacillus*. *Enterobacter* and *Clostridium* are undesirable bacteria in silage fermentation in that they would compete with LAB for nutrients and produce ammonia-N or butyric acid. Their principle fermentation product is not lactic acid but acetic acid or butyric acid, which is less efficient in pH decline and energy transition. Moreover, feeding animals with silage of substantial clostridial activity often leads to the decrease of feed intake, or causes ketosis (Muck, 2010). Even worse, the endotoxins of some species might affect animal performance and initiate food safety issue (Dunière et al., 2013). Accordingly, the dominance of these bacteria would mainly account for the poor fermentation quality of stylo silage. Meanwhile, the initiators of pH decline like *Lactobacillus, Lactococcus, Enterococcus, Weissella* had a relatively low abundance, inferring that LAB failed to establish the dominance in the stylo silage.

In comparison, TPP addition led to the decrease of the abundance of *Lactobacillus, Clostridium_sensu_stricto_1* and *Lachnoclostridium_5* as well as the total relative abundance of common LAB in silage, along increased *Pantoea* abundance. The most important species of *Clostridium* in silage are proteolytic clostridia, the *Clostridium butyricum* group and *Clostridium tyrobutyricum*, different in fermentation substrates (Muck, 2010). *Lachnoclostridium*, a newly proposed genus within the family Lachnospiraceae, a group of gram-positive, motile, obligately anaerobic spore-forming and rod-shape Clostridia, are able to ferment mono- and disaccharides mainly producing acetate (Yutin and Galperin, 2013). However, their roles in silage fermentation have been rarely reported so far. Based on their phyletic classification, they might function like Enterobacter or Clostridia but be somewhat different. Thus, their decreased abundance might partly explain the decrease of DM loss and ammonia-N in the treatment groups. Consistently, the increased abundance of *Pantoea* might contribute to the decrease of ammonia-N given that Ogunade et al. (2018) reported *Pantoea* abundance is negatively related to silage ammonia-N concentrations. The decreased relative abundance of common LAB was not consistent with the result of plate counting that TPP addition increased LAB population, inferring that TPP addition might boost the proliferation of silage microorganisms resulting in a larger biomass population. All these results suggest that LAB could not dominate the stylo silage during ensiling thus resulting in poor silage quality, and TPP addition exerts an effect on the bacterial community but not enough.

## Conclusions

From the above, considerable DM loss and proteolysis occurred in stylo silage, which could be reduced by TPP addition, particularly at the early stage of ensiling fermentation. Moreover, TPP addition altered the bacterial community of stylo silage, but the silage was still dominated by *Enterobacter* and *Clostridium*, and the decrease of *Clostridium_sensu_stricto_1* might primarily contribute to silage improvement. It is suggested that TPP application could help improve the fermentation quality and nutrient preservation of stylo silage, but the optimum dose still need further research.

## Data availability statement

The datasets presented in this study can be found in online repositories. The names of the repository/repositories and accession number(s) can be found in the article/supplementary material.

## Author contributions

YH: conceptualization, methodology, and writing and editing. CQ: investigation, data curation, and reviewing. YW: methodology, investigation, and formal analysis. WZ: validation and supervision. LH: resources, conceptualization, methodology, and visualization. All authors contributed to the article and approved the submitted version.

## Funding

This work got the financial support from China Agricultural Research System of MOF and MARA (CARS-39), and National Natural Science Foundation of China (Grant No. 32102562).

## Conflict of interest

The authors declare that the research was conducted in the absence of any commercial or financial relationships that could be construed as a potential conflict of interest.

## Publisher's note

All claims expressed in this article are solely those of the authors and do not necessarily represent those of their affiliated organizations, or those of the publisher, the editors and the reviewers. Any product that may be evaluated in this article, or claim that may be made by its manufacturer, is not guaranteed or endorsed by the publisher.
